# A simple analytical method for the simultaneous determination of multiple organic pollutants in sediment samples

**DOI:** 10.1016/j.mex.2018.08.012

**Published:** 2018-09-10

**Authors:** Xiangying Zeng, Qiongpu Hu, Lixiong He, Zhiyang Liu, Shutao Gao, Zhiqiang Yu

**Affiliations:** a State Key Laboratory of Organic Geochemistry, Guangdong Key Laboratory of Environmental Protection and Resources Utilization, Guangzhou Institute of Geochemistry, Chinese Academy of Sciences, Guangzhou 510640, China; b University of Chinese Academy of Sciences, Beijing 100049, China; c Fujian Provincial Academy of Environmental Science, Fuzhou 350000, China; d Institute of Atmospheric Environment, Guangdong Provincial Academy of Environmental Science, Guangzhou 510000, China

**Keywords:** Multiple-residue analytical method, Synthetic musks, PAHs, Organophosphate esters, UV filters, Analytical method

## Abstract

Due to the extensive application of synthetic organic chemicals, the resulting environmental contamination with such chemicals is of great concern. Herein, we describe the development of a simple analytical method to determine several groups of organic compounds in sediment samples. Samples were soxhlet-extracted with dichloromethane, separated, and cleaned-up by passage through a combined column of neutral alumina/silica gel, then identified and determined by GC—MS analysis. Four sediment samples were analyzed to validate the efficiency, and acceptable recoveries and good repeatability were obtained.

• Combined chromatographic columns of silica gel and alumina have been used for separation and clean-up.
• Five groups of organic compounds have been simultaneously analyzed.
• Acceptable recoveries with good reproducibility have been achieved.

## Graphical abstract

**Figure:**
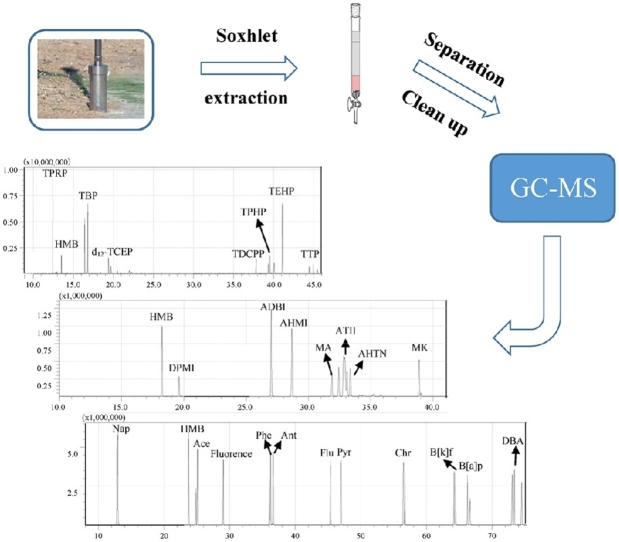

**Specifications Table**

**Table:** 

**Subject Area**	*Environmental Science*
**More specific subject area:**	*Analytical method*
**Method name:**	*Multiple-residue analytical method*
**Name and reference of original method**	Warren, N., Allan, I. J., Carter, J. E., House, W. A., Parker, A. 2003. Pesticides and other micro-organic contaminants in freshwater sedimentary environments – a review. Applied Geochemistry, 18(2): 159–194. Luo, Y., Guo, W., Ngo, H., Nghiem, L., Hai, F., Zhang, J., Liang, S., Wang, X. 2014. A review on the occurrence of micropollutants in the aquatic environment and their fate and removal during wastewater treatment. Science of The Total Environment, 473–474: 619–641. Bao, L., Maruya, K., Snyder, S., Zeng, E. Y. 2012. China’s water pollution by persistent organic pollutants. Environmental Pollution. 163: 100–108.
**Resource availability**	

## Introduction

The extensive usage of synthetic chemicals has resulted in their ubiquitous presence in every corner of the world, and due to their toxicities to humans and organisms, some of them are identified as emerging organic pollutants [4]. Simultaneously, some organic compounds unintentionally derived from precursors are also ubiquitous in the natural environment, such as polycyclic aromatic hydrocarbons (PAHs), which mainly originate from vehicular emissions and biomass/coal combustion [7]. The presence of traditional persistent organic pollutants (POPs) and emerging organic pollutants in the environment poses a great threat to human health and aquatic systems. Consequently, there is great concern about their occurrence and distribution in the environment. As reported in some reviews (Larivière et al., 2017; [5]), extensive studies have been carried out focusing on different classes of contaminants based on the development of specific analytical methods, but these have been limited to organic pollutants originating from different sources. In the present study, several groups of contaminants widely detected in sediment in the Pearl River Delta were chosen as target compounds, including PAHs, organochlorine pesticides (OCPs, namely HCHs and DDTs), synthetic musks used as fragrance materials in domestic products and personal products, UV filters used in personal products and consumer goods, as well as organophosphate esters (OPs) used as flame-retardants and plasticizers.

In order to understand their co-occurrence, possible ecological risk, and ultimate fate in the environment, simple and effective analytical methods are highly desirable. This study is aimed at optimizing and validating a sensitive method for the simultaneous determination of the abovementioned organic pollutants in sediment. Furthermore, to prove the applicability and robustness of the method, four sediment samples have been analyzed.

## Chemicals and materials

Five groups of organic chemicals were chosen as target compounds, comprising 16 PAHs, 7 OCPs, 9 synthetic musks, 5 UV filters, and 7 OPs.

Specifically, 16 PAHs listed as priority pollutants by the U.S. EPA were obtained from Supelco (Bellefonte, PA, USA), including naphthalene (Nap), acenaphthylene (Acy), acenaphthene (Ace), fluorene (Flu), phenanthrene (Phe), anthracene (Ant), fluoranthene (Fla), pyrene (Pyr), benzo[*a*]anthracene (BaA), chrysene (Chr), benzo[*b*]fluoranthene (BbF), benzo[*k*]fluoranthene (BkF), benzo[*a*]pyrene (BaP), dibenzo[*a*,*h*]anthracene (DahA), indeno[1,2,3-*cd*]pyrene (InP), and benzo[*g*,*h*,*i*]perylene (BghiP).

DDTs and HCHs were purchased from Supelco (Bellefonte, PA, USA).

Synthetic musks were purchased from LGC Promochem GmbH (Mercatorstrasse, Wesel, Germany), including Cashmeran (1,2,3,5,6,7-hexahydro-1,1,2,3,3-pentamethyl-4*H*-inden-4-one, DPMI), Celestolide (4-acetyl-1,1-dimethyl-6-*tert*-butylindane, ADBI), Phantolide (6-acetyl-1,1,2,3,3,5-hexamethylindane, AHMI), Traseolide (5-acetyl-1,1,2,6-tetramethyl-3-isopropylindane, ATII), Tonalide (6-acetyl-1,2,3,4-tetrahydro-1,1,2,4,4,7-hexamethylnaphthalene, AHTN), Galaxolide (1,3,4,6,7,8-hexahydro-4,6,6,7,8,8-hexamethylcyclopenta-2-benzopyran, HHCB), Musk ketone (4-*tert*-butyl-2,6-dimethyl-3,5-dinitroacetophenone, MK), Musk ambrette (1-(*tert*-butyl)-2-methoxy-4-methyl-3,5-dinitrobenzene, MA), and Musk xylene (1-*tert*-butyl-3,5-dimethyl-2,4,6-trinitrobenzene, MX).

Five UV filters were procured from AccuStandard (New Haven, CT, USA), namely octocrylene (OC), ethylhexyl methoxycinnamate (EHMC), ethylhexyl dimethyl *p*-aminobenzoate (ODPABA), 4-methylbenzylidene camphor (4-MBC), and benzophenone (BP).

Seven OP standards were purchased from Sigma-Aldrich (St. Louis, MO, USA), namely tris(2-butoxyethyl) phosphate (TBEOP), tributyl phosphate (TNBP), triphenyl phosphate (TPhP), tritolyl phosphate (TMPP), tris(2-chloroethyl) phosphate (TCEP), tris(2-chloropropyl) phosphate (TCIPP), and tris(1,3-dichloropropyl) phosphate (TDCIPP).

Five deuterated PAHs (d_8_-Nap, d_10_-Ace, d_10_-Phe, d_12_-Chr, and d_12_-Perylene (d_12_-Per)) were also obtained from Supelco (Bellefonte, PA, USA); d_27_-TNBP, d_15_-TPHP, d_12_-TCEP, and d_15_-MX were obtained from C/D/N Isotopes Inc. (Quebec, Canada). These deuterated chemicals were used as surrogate standards. The internal standard hexamethylbenzene (HMB) was obtained from the laboratories of Ehrenstofer-Schäfer Bgm-Schlosser (Augsburg, Germany).

All solvents used in this study were of chromatographic grade. Methanol (MeOH) was purchased from J&K Chemical (Beijing, China). Dichloromethane (CH_2_Cl_2_), ethyl acetate (EtOAc), *n*-hexane (Hex), and Acetone (Acet) were purchased from CNW Technologies GmbH (Düsseldorf, Germany).

Silica gel (70–230 mesh) was obtained from Merck Co. (Darmstadt, Germany), activated at 180 °C for 12 h, deactivated with 3% redistilled water, and kept in *n*-hexane before use. Neutral alumina (100–200 mesh, Shanghai Wusi Chemical Reagent Co., China) was continuously soxhlet-extracted with MeOH and CH_2_Cl_2_ for 48 h, then activated at 250 °C for 12 h, deactivated with 3% redistilled water, and kept in *n*-hexane prior to use. Anhydrous Na_2_SO_4_ was placed in a furnace at 450 °C for 4–6 h before use.

## Extraction, separation, and purification

Approximately 10 g of Dry sediment samples, spiked with 50 ng surrogate standards (d8-Nap, d10-Ace, d10-Phe, d12-Chr, d12-Per, d27-TNBP, d15-TPHP, d12-TCEP and d15-MX), and then soxhlet-extracted with CH_2_Cl_2_ (200 mL) for 72 h. Activated copper was used to remove elemental sulfur potentially present in the sediment samples. The extracts were concentrated with a rotary evaporator to a volume of about 1 mL, then the solvent was exchanged into *n*-hexane, and concentrated to approximately 1 mL finally.

A glass chromatographic column (0.8 cm i.d. × 30 cm), fitted with a Teflon stopcock, was packed consecutively with neutral alumina and silica gel (1:2) and anhydrous Na_2_SO_4_. The final extracts were loaded onto the chromatographic column for further separation and clean-up. Mixed solvents with different polarity were used to elute and recover the target compounds. Firstly, *n*-hexane (15 mL) was used to transfer the final extract onto the chromatographic column and discarded due to no target compounds being present in this fraction. Fraction 1 was then collected in *n*-hexane/CH_2_Cl_2_ (3:1, v/v; 50 mL), eluting PAHs, DDTs, HCHs, and two nitro musks (MA and MX); Fraction 2 was collected in *n*-hexane/CH_2_Cl_2_ (1:1, v/v; 60 mL) for polycyclic musks, MK, and UV filters; Fraction 3 was collected in EtOAc (100 mL) for OPs.

## Instrumental analyses

The collected fractions were concentrated to a volume of about 1 mL, solvent-exchanged into *n*-hexane, and further concentrated to 200 μL under a gentle N_2_ stream. An aliquot of 100 ng internal standard (HMB) was added before instrumental analysis.

Identification and determination of target compounds were performed using a Shimadzu GC–MS-QP 2010 (Shimadzu, Kyoto, Japan) in electron impact (EI) ionization mode with helium as the carrier gas at a flow rate of 1 mL/min. The injection was performed in splitless mode at 290 °C. The mass spectrometer was operated in selected-ion mode under EI, and the source temperature and transfer line were set at 220 °C and 300 °C, respectively.

For GC separation, a TG-5MS column (30 m × 0.25 mm i.d. × 0.25 μm film) (Thermo Fisher Scientific, Waltham, MA, USA) was used for OPs, and a DB-5MS column (30 m × 0.25 mm i.d. × 0.25 μm film) (J & W Scientific Co. Ltd., Folsom, CA, USA) was used for PAHs, OCPs, UV filters, and SMs.

The temperature programs for the different compounds were as follows:

$$\text{PAHs}:70{}^{\circ}\text{C}\;\left(2\;\text{min}\right)\overset{3{}^{\circ}C/\text{min}}{\to }295{}^{\circ}\text{C}\;\left(5\;\text{min}\right);$$

$$\text{OCPs}:90{}^{\circ}\text{C}\;\left(1\;\text{min}\right)\overset{10{}^{\circ}\text{C/min}}{⟶}180{}^{\circ}\text{C}\;\left(2\;\text{min}\right)\overset{4{}^{\circ}\text{C/min}}{⟶}250{}^{\circ}\text{C}\overset{10{}^{\circ}\text{C/min}}{⟶}300{}^{\circ}\text{C}\left(5\;\text{min}\right);$$

$$\text{Synthetic}\;\text{musks}:70{}^{\circ}C\;\left(2\;\text{min}\right)\overset{5{}^{\circ}C/\text{min}}{⟶}150{}^{\circ}C\overset{1.5{}^{\circ}C/\text{min}}{⟶}180{}^{\circ}C\;\overset{10{}^{\circ}C/\text{min}}{⟶}\;295{}^{\circ}C\;(5\;\text{min}$$

$$\text{UV}\;\text{filters}:70{}^{\circ}C\;\left(2\;\text{min}\right)\overset{10{}^{\circ}C/\text{min}}{⟶}190{}^{\circ}C\;\left(8\;\text{min}\right)\overset{1{}^{\circ}C/\text{min}}{⟶}200{}^{\circ}C\overset{10{}^{\circ}C/\text{min}}{⟶}300{}^{\circ}C\left(8\;\text{min}\right)$$

$$\text{OPs}:\;70{}^{\circ}C\;\left(2\;\text{min}\right)\overset{10{}^{\circ}C/\text{min}}{⟶}160{}^{\circ}C\overset{5{}^{\circ}C/\text{min}}{⟶}295{}^{\circ}C\;\left(10\;\text{min}\right)$$

## Quality assurance/quality control

Recovery studies were performed with mixtures of target compounds in a solvent. For this purpose, *n*-hexane was fortified with 50 ng mixtures of standards, which were separated on the combined chromatographic column. The recovery values were then calculated by dividing the obtained concentration by the theoretical concentration calculated from the added amounts. Six replicate samples were analyzed, and the recoveries for the target compounds are listed in Table 1.

**Table 1:** The recoveries and RSDs (*n* = 6) of the target compounds.

Compounds	Recoveries/%	RSD/%
PAHs		
Ace	92.9	11.0
Dih	94.5	11.0
Flu	93.5	11.0
Phe	100.5	9.0
Ant	87.8	7.3
Fla	100.7	9.9
Pyr	99.7	9.2
BaA	104.2	2.3
Chr	106.9	0.8
BbF	108.4	2.3
BkF	107.1	1.2
BaP	98.7	1.8
Ind	104.9	5.0
BghiP	106.3	7.4
Dib	105.0	6.2

OCPs		
α-HCH	105.5	9.4
β-HCH	103.4	9.7
γ-HCH	111.8	10.8
δ-HCH	106.8	15.0
DDT	63.5	16.2
DDD	94.9	12.8
DDE	109.4	13.1

Synthetic musks		
DPMI	94.1	13.1
ADBI	96.3	7.3
AHMI	98.4	6.2
ATII	97.2	5.7
HHCB	96.1	4.2
AHTN	99.9	5.9
MK	99.9	2.4
MA	87.7	10.2
MX	86.6	8.9

UV Filters		
BP	92.8	2.6
4MBC	101.9	6.7
ODPABA	106.3	10.0
EHMC	106.9	23.8
OC	118.2	4.8

OPs		
TNBP	83.0	6.5
TBEOP	126.1	3.4
TCEP	102.2	3.4
TCIPP	94.5	3.3
TDCIPP	93.0	5.3
TPhP	72.0	3.8
TMPP	70.2	6.2

In addition, blanks (*n* = 6) were analyzed as real samples. The results indicated that TCEP, TCIPP, and HHCB were detected in all of the blanks below the limits of quantification (LODs), whereas the other target compounds were not found in any of the blanks.

The LODs were calculated as 3× (S.D./S), where S.D. is the standard deviation of the response acquired for seven replicate injections of standards at concentrations as low as possible, which was established using dilution series of a stock solution of each compound. The limits of quantification (LOQs) were calculated as twice the LODs [2]. Based on the developed analytical method, acceptable results have been achieved in terms of LODs in 10 g of sediment for PAHs (0.09–0.10 ng/g), OCPs (0.08–0.20 ng/g), synthetic musks (0.03–0.04 ng/g), UV filters (0.16–0.33 ng/g), and OPs (0.06–0.18 ng/g).

## Validation of the analytical method

To evaluate the effectiveness of the established analytical method, it was applied to four sediment samples. Meanwhile, QA/QC samples, including blanks (solvent, *n* = 3), spiked blanks (spiked standards in solvent, *n* = 3), and spiked matrix (standards spiked into pre-extracted sediment, *n* = 3) were analyzed as real sediment samples.

Due to its high volatility, recovery of d_8_-Nap was not calculated, and Nap was not quantified in the present study. Acceptable recoveries of the other surrogates were achieved with good reproducibility. Specifically, the recoveries of these surrogates were as follows: d_10_-Ace (74.8%±12.1%), d_10_-Phe (92.3%±8.7%), d_12_-Chr (66.7%±11.8%), d_12_-Per (65.8%±12.4%), d_27_-TBP (120.4%±6.8%), d_12_-TECP (118.1 ± 13.3%), d_15_-TPhP (88.7 ± 12.1%), and d_15_-MX (92.4 ± 8.5%).

The results indicated that most of the target compounds were found in the sediment samples. All 16 PAHs were detected in the sediment, with ∑15PAHs varying in the range 2145.2–2533.2 ng/g; HCHs and DDTs were found at levels of 0.06–0.95 ng/g and 0.19–2.78 ng/g, respectively. Meanwhile, HHCB and AHTN were measured at concentrations of 1.03–1.69 ng/g and 1.25–1.70 ng/g, respectively, but MX was found to be below the LOD, and the other synthetic musks were not found in any of the four sediments. The results agreed to the current market information which HHCB and AHTN were predominate over the fragrance materials. As for the five UV filters, BP (6.26–10.43 ng/g), OC (0.66–0.97 ng/g), and 4MBC (0.50–0.85 ng/g) were found in all four sediments, and EHMC (0.14–0.69 ng/g) was found in three of the sediments. However, ODPABA was not detected in any of the samples. The OPs were found in all of the samples at different concentrations. For example, TBEOP was found below the LOD, TBP at 3.39–3.75 ng/g, TPhP at 1.55–3.17 ng/g, and TMPP at 1.45–2.34 ng/g. Simultaneously, three chlorinated OPs, TECP (1.68–23.41 ng/g), TCIPP (1.46–7.78 ng/g), and TDCIPP (3.77–12.26 ng/g), were found in the samples.

## Conclusion

Based on soxhlet extraction coupled with separation/clean-up on a chromatographic column packed with neutral alumina and silica gel, a simple and effective analytical method has been developed for the simultaneous determination of five groups of organic pollutants, namely PAHs, DDTs and HCHs, synthetic musks, UV filters, and OPs. The developed analytical method exhibited good recoveries with excellent reproducibilities. By integrating the analytical process and operation, we can obtain more information about the co-occurrence of common contaminants in sediment, using limited samples with less consumption of energy, reagents, time, and manpower.
